
JOURNAL INFORMATION
==============================
NLM Title Abbreviation: Rheumatol Adv Pract
Journal ID: Rheumatol Adv Pract
NLM Journal ID: 101736676
Journal ID: rheumap

Rheumatology Advances in Practice

EISSN: 2514-1775

ARTICLE INFORMATION
==============================
PMCID: PMC8194598
PMID: 34159291
DOI: 10.1093/rap/rkab035
Article ID: rkab035
Article version: 1
Subjects: Letter to the Editor (Case Report), AcademicSubjects/MED00010

A Kawasaki-like illness in an adult with recent SARS-CoV-2 infection

Nagra Deepak 1
Russell Mark D 1
Rosmini Stefania 2
Sado Daniel 2
Buazon April 1
Shafi Taimur 2
Hamlyn Elizabeth 2
Sandhu Gurjinder 2
Rutherford Andrew I 2
Galloway James B 1
1 Centre for Rheumatic Disease, King’s College London , London, UK
2 King’s College Hospital NHS Foundation Trust , London, UK
Corresponding author: Dr Deepak Nagra; Centre for Rheumatic Diseases, Weston Education Centre, King’s College London, 10 Cutcombe Road, London, SE5 9RJ, UK; deepak.nagra@nhs.net
Electronic publication date: 2021 May 18
Electronic Location ID: rkab035
Received 2021 Apr 21; Accepted 2021 May 10
Copyright: © The Author(s) 2021. Published by Oxford University Press on behalf of the British Society for Rheumatology.
Copyright year: 2021
License: This is an Open Access article distributed under the terms of the Creative Commons Attribution License (http://creativecommons.org/licenses/by/4.0/), which permits unrestricted reuse, distribution, and reproduction in any medium, provided the original work is properly cited.
License URL: https://creativecommons.org/licenses/by/4.0/

Page count: 7
edited-state: accepted-manuscript
article-lifecycle: PAP

==============================
### A Kawasaki-like illness in an adult with recent SARS-CoV-2 infection

### Authors

Deepak Nagra[1], Mark D. Russell[1], Stefania Rosmini[2], Daniel Sado[2], April Buazon[1], Taimur Shafi[2], Elizabeth Hamlyn[2], Gurjinder Sandhu[2], Andrew I. Rutherford[2], James B. Galloway[1]

### Affiliations

1. Centre for Rheumatic Disease, King's College London, London, UK
2. King's College Hospital NHS Foundation Trust, London, UK

### Corresponding author

Dr Deepak Nagra; Centre for Rheumatic Diseases, Weston Education Centre, King's College London, 10 Cutcombe Road, London, SE5 9RJ, UK; deepak.nagra@nhs.net

© The Author(s) 2021. Published by Oxford University Press on behalf of the British Society for Rheumatology.
This is an Open Access article distributed under the terms of the Creative Commons Attribution License (http://creativecommons.org/licenses/by/4.0/), which permits unrestricted reuse, distribution, and reproduction in any medium, provided the original work is properly cited. For commercial re-use, please contact journals.permissions@oup.com

**Key message:** Kawasaki-like illnesses are rare but important diagnoses to consider in adults with recent COVID-19.

Dear Editor,

Paediatric multisystem inflammatory syndrome temporally associated with SARS-CoV-2 (PIMS-TS) is an increasingly recognised entity, with features of both Kawasaki disease and toxic shock syndrome. There have been multiple reports of this syndrome in the paediatric population(1, 2). We describe an adult male presenting with features suggestive of Kawasaki disease following recently confirmed infection with SARS-CoV-2.

A 30-year-old man of Sri Lankan ethnicity presented to our Emergency Department (ED) one month after PCR-confirmed COVID-19. His initial COVID-19 symptoms had been cough, fevers and fatigue, with resolution of these symptoms two weeks after onset. Subsequent PCR tests for SARS-CoV-2 were negative.

At presentation to our ED, the patient reported 7-days of headaches, fevers, abdominal pain and vomiting. He had no other localising symptoms, including no cardiorespiratory symptoms. He was previously fit and well, and he did not take any medications. He had moved to the United Kingdom from Sri Lanka in 2015. There was no recent history of travel or known infectious contacts.

At presentation, he had a heart rate of 123 bpm, respiratory rate of 22 breaths per minute, blood pressure of 90/53 mmHg, oxygen saturations of 99% on air, and temperature of 38.0°C. His GCS was 15/15. Examination revealed bilateral conjunctival injection (Figure 1A) and cracking of the lips (Figure 1B). Full systems examination was otherwise unremarkable, including no evidence of a rash or palpable lymphadenopathy.

Blood tests at presentation showed: white cell count 15.1 $x10^9$/L, neutrophils count 13.5 $x10^9$/L, lymphocyte count 0.96 $x10^9$/L, haemoglobin 154 $x10^9$/L, platelet count 248 $x10^9$/L, CRP 396 mg/L, sodium 128 mmol/L, creatinine 103 micromol/L, aspartate transaminase 48 IU/L, alanine phosphatase 117 IU/L. There were elevations in Troponin T (259 ng/L), D-dimer (3620 ng/ml), NT-pro-BNP (911 ng/L) and ferritin (2609). Anti-nuclear antibodies, anti-double stranded DNA and anti-neutrophil cytoplasmic antibodies were negative, and there was no evidence of complement consumption. Tests for HIV, hepatitis A/B/C, Epstein-Barr virus, cytomegalovirus, leptospirosis, syphilis, rubella and parvovirus were negative. Blood and urine cultures were sterile. SARS-CoV-2 PCR was negative on admission to the ED, a chest radiograph was normal, while SARS-CoV-2 IgG was positive. A 12-lead ECG showed sinus rhythm with T-wave inversion in leads III and V3-V5.

Despite broad-spectrum, intravenous antibiotics and fluid resuscitation, the patient remained persistently febrile and hypotensive. CT pulmonary angiography showed no pulmonary emboli but demonstrated mediastinal lymphadenopathy and small bilateral pleural effusions. A cardiac MRI demonstrated a small localised pericardial effusion, preserved left ventricular function, focal hypokinesia at the mid inferolateral wall and features of active myocardial inflammation (Figure 1C): high native myocardial T1 signal at the basal to mid inferolateral wall (1185ms; normal values, 950-1100ms using MOLLI sequence at 1.5T) and matched elevated T2 signal (58ms; normal values <50ms) with normal T1 and T2 values in the remote septal myocardium.

A rheumatology opinion was sought; the constellation of persistent fever, bilateral conjunctival injection, mucosal changes and myocarditis were suggestive of a Kawasaki-like illness. Intravenous immunoglobulin (IVIg) at 2 g/kg was administered over two days, in addition to oral aspirin 900 mg daily. The patient responded well, with rapid normalisation of his blood pressure, temperature and other symptoms. He was discharged home on aspirin and ramipril for cardiovascular protection, and continues to do well. Four weeks after discharge from hospital, the patient noted desquamation of the palms of both hands. CT coronary angiography and repeat cardiac MRI are scheduled.

In this report, we describe the successful treatment of an adult with a Kawasaki-like illness temporally associated with SARS-CoV-2 infection. Many of the features in this case mimic those seen in reports of PIMS-TS, including the gastrointestinal symptoms, conjunctival injection, desquamation, myocarditis and cardiogenic shock at presentation(1, 2). Our case meets the criteria selected by the Centres for Disease Control and Prevention (CDC) in their report of 27 cases of multisystem inflammatory syndromes in adults associated with SARS-CoV-2 infection: age ≥21 years; current or recently-confirmed infection with SARS-CoV-2; severe extra-pulmonary organ dysfunction in the absence of severe respiratory illness; and laboratory evidence of severe inflammation(3). Of note, 63% of cases in the CDC's report were positive for SARS-CoV-2 at the time of presentation, with typical chest imaging features of COVID-19. It is therefore plausible that direct viral injury may have contributed to many of the presentations in the CDC's report, reflective of the broad inclusion criteria.

In this case, the onset of symptoms one month after the initial SARS-CoV-2 infection, coupled with the negative SARS-CoV-2 PCR test on presentation to the emergency department, is strongly suggestive of an immunological-mediated process rather than direct viral insult. Recent national consensus guidelines for PIMS-TS separate the presenting phenotype into Kawasaki disease-like presentations and non-specific presentations that do not meet criteria for Kawasaki disease(4), with the recommended first-line treatment being IVIg. Adopting a similar approach for multisystem inflammatory syndrome in adults with recent COVID-19 seems pragmatic. This case highlights the ability of SARS-CoV-2 to trigger unusual immunological adverse events, and the rheumatology community need to be vigilant for these complications.

**Funding**

No specific funding was received from any bodies in the public, commercial or not-for-profit sectors to carry out the work described in this article.

**Conflict of interest**

JG receives speaker fees from Abbvie, Biovitrum, BMS, Celgene, Chugai, Gilead, Janssen, Lilly, Novartis, Pfizer, Roche, Sanofi, Sobi and UCB. MDR has received honoraria from Pfizer, Lilly and UCB. The other authors have declared no conflicts of interest.

**References**

1. Riphagen S, Gomez X, Gonzalez-Martinez C, Wilkinson N, Theocharis P. Hyperinflammatory shock in children during COVID-19 pandemic. Lancet. 2020;395(10237):1607-8.

2. Davies P, Evans C, Kanthimathinathan HK, Lillie J, Brierley J, Waters G, et al. Intensive care admissions of children with paediatric inflammatory multisystem syndrome temporally associated with SARS-CoV-2 (PIMS-TS) in the UK: a multicentre observational study. Lancet Child Adolesc Health. 2020;4(9):669-77.

3. Morris SB, Schwartz NG, Patel P, Abbo L, Beauchamps L, Balan S, et al. Case Series of Multisystem Inflammatory Syndrome in Adults Associated with SARS-CoV-2 Infection - United Kingdom and United States, March-August 2020. MMWR Morb Mortal Wkly Rep. 2020;69(40):1450-6.

4. Harwood R, Allin B, Jones CE, Whittaker E, Ramnarayan P, Ramanan AV, et al. A national consensus management pathway for paediatric inflammatory multisystem syndrome temporally associated with COVID-19 (PIMS-TS): results of a national Delphi process. Lancet Child Adolesc Health. 2021;5(2):133-41.

**Figure 1. Conjunctival and oral manifestations and cardiac MRI findings in a Kawasaki-like illness post-COVID-19.**

**A:** Bilateral conjunctival injection; **B:** Cheilitis at presentation; **C**: Cardiac MRI demonstrating features of active myocardial inflammation, with high native myocardial T1 signal at the basal to mid inferolateral wall (left image) and matched elevated T2 signal (right image).

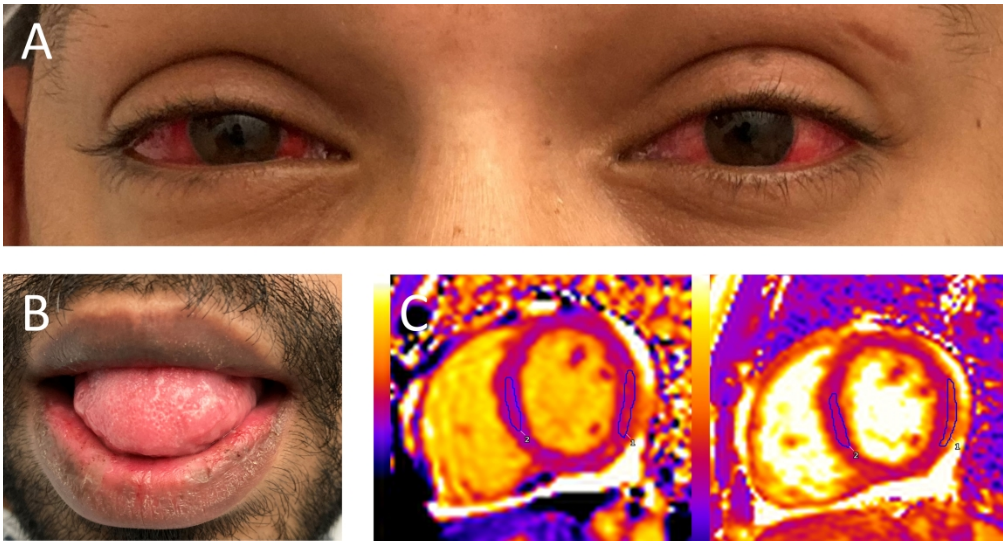

Figure 1. Conjunctival and oral manifestations and cardiac MRI findings in a Kawasaki-like illness post-COVID-19.
A: Bilateral conjunctival injection; B: Cheilitis at presentation; C: Cardiac MRI demonstrating features of active myocardial inflammation, with high native myocardial T1 signal at the basal to mid inferolateral wall (left image) and matched elevated T2 signal (right image).

337x181mm (300 x 300 DPI)
